# Psychometric Properties of the Serbian Teen Version of the Problem Areas in Diabetes Scale—A Validation Study

**DOI:** 10.3390/nursrep15090326

**Published:** 2025-09-08

**Authors:** Mirjana Smudja, Tatjana Milenković, Ivana Minaković, Vera Zdravković, Sandra Mitić, Ana Miljković, Dragana Milutinović

**Affiliations:** 1 Faculty of Medicine, University of Novi Sad, Hajduk Veljkova 3, 21000 Novi Sad, Serbia; ivana.minakovic@mf.uns.ac.rs (I.M.); ana.miljkovic@mf.uns.ac.rs (A.M.); 2 Department of Higher Medical School, Academy for Applied Studies Belgrade, Cara Dusana 254, 11080 Belgrade, Serbia; 3 Department of Endocrinology, Mother and Child Health Care Institute of Serbia “Dr. Vukan Cupic”, Radoja Dakica 6-8, 11070 Belgrade, Serbia; tanjamil5e@gmail.com; 4 Health Center Novi Sad, Bulevar Cara Lazara 75, 21000 Novi Sad, Serbia; 5 University Children’s Hospital Belgrade, Tirsova 10, 11000 Belgrade, Serbia; vera.zdravkovic@udk.bg.ac.rs; 6 Faculty of Medicine, University of Belgrade, Dr. Subotica 8, 11000 Belgrade, Serbia; 7 Specialized Hospital “Bukovicka Banja” Arandjelovac, Misarska 1, 34300 Arandjelovac, Serbia; s.mitic@bukovickabanja.co.rs; 8 Department of Nursing, Faculty of Medicine, University of Novi Sad, Hajduk Veljkova 3, 21000 Novi Sad, Serbia

**Keywords:** adolescents, type 1 diabetes, diabetes distress, nursing, scale validation, reliability, psychometrics

## Abstract

Screening for diabetes-specific distress should be considered a standard component of diabetes management. This study aimed to evaluate the psychometric properties of the Serbian adaptation of the Problem Areas in Diabetes—Teen Version (PAID-T). **Methods:** A multicentre, validation, cross-sectional study was conducted with 374 adolescents (aged 13–18 years) diagnosed with type 1 diabetes (T1D), all of whom completed the Serbian version of the PAID-T. The psychometric evaluation included assessments of construct validity through exploratory (EFA, first subsample, *n* = 140) and confirmatory (CFA, second subsample, *n* = 234) factor analyses, as well as examinations of concurrent and convergent validity. Reliability was evaluated using measures of internal consistency and test–retest stability (*n* = 289). **Results:** Factor analyses indicated some multidimensionality; however, the high correlations between factors in the three-factor model and the optimal fit of the hierarchical three-factor model with a single second-order factor supported the interpretation that the PAID-T measures a unified construct, with satisfactory fit indices (CFI = 0.95; TLI = 0.93; RMSEA = 0.08; SRMR = 0.05). Concurrent validity testing demonstrated gender-based differences in adolescents’ perceptions of the emotional burden of diabetes (W = 19.718, *p* = 0.03, small effect size = 0.11). Convergent validity analyses showed that adolescents who were non-adherent to treatment (W = 11.390, *p* = 0.01, small effect size = 0.13) or experienced difficulties managing diabetes at school (W = 16.333, *p* < 0.001, small effect size = 0.16) reported significantly higher levels of diabetes-specific distress. A significant negative correlation was also observed between PAID-T scores and perceived social support (ρ = −0.24, *p* < 0.001). Importantly, Serbian adolescents with T1D reported mean PAID-T scores close to the cutoff point of 44, indicating clinically relevant levels of distress. The Serbian version demonstrated strong internal consistency (Cronbach’s α = 0.92; McDonald’s ω = 0.93) and excellent test–retest reliability (ICC = 0.99, 95% CI), confirming stability over time. **Conclusions:** The Serbian adaptation of the PAID-T demonstrated strong validity and reliability, supporting its use as a robust tool for assessing self-reported diabetes-specific distress in adolescents. Notably, the mean PAID-T scores in Serbian adolescents with T1D were close to the established cutoff point of 44, underscoring the clinical relevance of routine screening in this population. The early identification of diabetes distress can enable nurses and other members of the multidisciplinary healthcare team to deliver tailored interventions, ultimately improving psychological well-being and health outcomes.

## 1. Introduction

T1D represents the most common metabolic disorder in childhood, imposing a lifelong burden on both affected paediatric patients and their caregivers. The incidence and severity of T1D have been increasing over recent decades. T1D is prevalent among Serbian youths (aged 0–19 years), with a notable incidence rate of 16.4 per 100,000 individuals. Analysis of Serbia’s National Diabetes Registry reveals a substantial annual increase in newly diagnosed paediatric patients with T1D, particularly in the 5–9 years age group (17.1 per 100,000 individuals) and the 10–14 years age group (29.2 per 100,000 individuals) [1,2].

To date, numerous studies have emphasised the multifaceted interaction between immune dysregulation, genetic predisposition, and environmental triggers in the pathogenesis of T1D. The natural history of T1D is typically described in three stages: stage 1 involves the development of β-cell autoimmunity, characterised by normoglycemia and the absence of clinical symptoms; stage 2 is defined by the onset of dysglycaemia in the absence of overt symptoms; and stage 3 is characterised by symptomatic hyperglycaemia. The majority of individuals who eventually develop T1D seroconvert to islet autoantibody positivity during childhood. The diagnostic accuracy of islet autoantibodies is influenced by testing strategies that include multiple antibody types, typically three to four, such as insulin autoantibodies (IAA), glutamic acid decarboxylase-65 (GAD-65), insulinoma-associated antigen-2 (IA-2A), and zinc transporter 8 (ZnT8). Assessing a panel of different islet autoantibodies—most commonly IAA, GAD-65, IA-2A, and ZnT8—enhances both sensitivity and specificity when these markers are used as biomarkers. For instance, a single screening at age 4 years for multiple autoantibodies identifies approximately 40% of individuals who will be diagnosed with T1D by age 15, whereas repeated screening at ages 2 and 6 increases sensitivity to more than 80%. The early recognition of T1D in its presymptomatic stages has substantial clinical implications, enabling timely support for paediatric patients and their families and providing opportunities to initiate disease-modifying interventions. One such therapy, teplizumab, was approved in the United States on 17 November 2022, under the brand name Tzield. Its safety and efficacy are currently under evaluation by the European Medicines Agency (EMA) [3,4,5].

Advances in diabetes care, including continuous glucose monitoring (CGM), insulin pumps (IP), and, more recently, smart multiple-dose injection (MDI) pens, have substantially improved glycaemic control and quality of life in paediatric patients [3,6,7]. However, access to these technologies remains unequal across the globe, with children in low-income settings facing significant barriers to precision treatment [3,7]. Moreover, the effective use of diabetes technology requires considerable knowledge and skills from both adolescents and their caregivers, who must navigate complex information that directly influences insulin therapy. To maximise the benefits of these devices, ongoing support from the diabetes care team is often necessary [1,6]. The transition of diabetes management responsibilities from parents to adolescents further complicates this process, as it is frequently accompanied by increased emotional distress, anxiety, depressive symptoms [8,9,10,11,12,13], limited coping capacity and problem-solving skills [1,6,14], and, in many cases, suboptimal metabolic control [1,15,16,17,18].

The inadequate control of diabetes significantly increases the risk of developing serious complications—neuropathy, nephropathy, and retinopathy, as well as vascular damage—which can be accelerated, sometimes resulting in early mortality [1,19,20,21]. Monitoring adherence to self-care behaviours is, therefore, a crucial step in ensuring effective care and predicting clinical outcomes in both the short and long term. Addressing these challenges enhances adolescents’ perceived self-efficacy in managing diabetes [1,22,23,24] and reduces diabetes-specific distress [8,11].

Diabetes specialist nurses (DSNs) play a crucial role in empowering patients and ensuring the delivery of high-quality care. In the Serbian healthcare system, paediatric patients and their caregivers benefit from educational programmes designed to strengthen self-management competencies and improve health-related quality of life [18]. Through empowerment, adolescents can gain greater confidence in managing their condition and adhering to treatment regimens [22,23,24,25,26]. According to Bandura, self-efficacy refers to an individual’s assessment of their capability to plan and execute actions specific to particular tasks and contexts [24,25,26]. The existing literature indicates that self-efficacy is both an outcome and a precursor of empowerment [24,25,26,27,28]. The coronavirus disease 2019 (COVID-19) pandemic accelerated the global adoption of telemedicine, ensuring continuity of care and facilitating diabetes education and empowerment through multiple platforms, including smartphones, video and audio consultations, web-based services, text messaging, mobile applications, or a combination of these approaches [29]. Such communication channels are particularly familiar to adolescents, making them effective tools for supporting diabetes self-management.

Self-efficacy has been identified as a key determinant of effective diabetes management. According to Survonen et al. [24], higher self-efficacy is positively associated with a better understanding of diabetes and its treatment, the successful integration of diabetes management into daily life, and improved relationships with healthcare providers. Individuals with greater self-efficacy are more likely to engage in appropriate self-care practices [24,25,29,30]. Nevertheless, Survonen et al. [24] found no significant relationship between self-efficacy and metabolic control. Conversely, Mahjouri et al. [31] reported a slight negative association between HbA1c levels and diabetes empowerment scores, as well as a negative correlation between the Iranian versions of the DES-28 scale and the Problem Areas in Diabetes (PAID) questionnaire, which captures emotional difficulties related to living with diabetes in adults.

These findings highlight the importance of screening for diabetes-specific psychosocial concerns as an integral part of care. Key issues include diabetes distress [29,32], depressive symptoms, and anxiety [32,33], all of which may significantly influence glycaemic control. While diabetes distress shares certain features with depression, it constitutes a distinct construct. Although not uniformly defined, diabetes distress is typically characterised by negative emotions and concerns specifically related to managing diabetes, such as frustration with persistent hyperglycaemia, feelings of inadequate support from family or peers, or the sense of being overwhelmed by the demands of treatment. Numerous studies report that approximately one-third of adolescents with T1D experience clinically relevant levels of diabetes distress, underscoring its strong association with the daily challenges of managing this complex chronic condition [34,35,36,37,38,39,40,41]. Notably, research by Hagger et al. [8] demonstrated that diabetes distress is more strongly correlated with HbA1c levels than depressive symptoms in adolescents with T1D. Consequently, the International Society for Pediatric and Adolescent Diabetes (ISPAD) recommends routine screening and assessment for diabetes-specific distress in adolescents both at diagnosis and during follow-up care [36]. Addressing diabetes distress is essential, given its significant impact on self-care behaviours, glycaemic outcomes, and psychosocial well-being [8,29,32,37,38]. The accurate assessment of the scope and nature of this burden is critical for developing targeted empowerment interventions for adolescents and their families [32,34].

The Problem Areas in Diabetes—Teen version (PAID-T) is one of the most widely used instruments for evaluating diabetes-related distress in adolescents [13,28,34,37]. The original PAID-T consisted of 26 items; however, in 2011, Weissberg-Benchell and Antisdel-Lomaglio [13] employed EFA to support a unidimensional construct and proposed a shortened 14-item version in identifying distress among adolescents with diabetes. In 2018, Shapiro et al. [34] further validated the 14-item PAID-T and identified three core constructs—emotional burden, family and friends distress, and regimen-specific distress—which together explained 64.3% of the total variance. CFA demonstrated an acceptable fit of the three-factor model (SRMR = 0.06; CFI = 0.98; RMSEA = 0.08). The total score of the 14-item PAID-T showed excellent internal consistency (Cronbach’s α = 0.93). Moreover, Shapiro et al. suggested a cutoff score of 44 or higher to indicate clinically significant distress in adolescents [34].

Additional support for the three-factor structure was provided by a German validation study, which replicated the findings of Shapiro et al. In this study, Cronbach’s α for the 14-item PAID-T was also high (α = 0.91). Item-to-total correlations ranged from 0.53 to 0.69, and the removal of any item did not improve internal consistency, thereby confirming the robustness of the PAID-T in German-speaking populations [37].

Although previous studies consistently emphasise the clinical importance of addressing diabetes-related distress [34,37,38,40] and strengthening self-efficacy [24,29] to improve self-management and facilitate the transition to adult care, the psychometric properties of the 14-item PAID-T have so far only been examined in English [34] and German [37].

At present, emotional problems in Serbian adolescents with T1D are primarily assessed using generic depression scales that do not adequately capture the disease-specific context. Given the absence of a validated version of the PAID-T in Serbian or other South Slavic languages (e.g., Croatian, Bosnian, Montenegrin, Slovenian, Bulgarian, Macedonian), the primary objective of this study was to translate the 14-item PAID-T questionnaire into Serbian (SRB_PAID-T 14) and to evaluate its construct validity, concurrent validity, and convergent validity. The secondary objective was to assess the instrument’s reliability by examining internal consistency and test–retest reliability.

The research question: Is the PAID-T questionnaire as accurate and dependable as a measurement tool for diabetes distress among adolescents with T1D in the Serbian-speaking community?

**Hypothesis** **1.** *We hypothesise that the Serbian version of the PAID-T will demonstrate sufficient construct validity. Specifically, we expect to identify three factors as the core constructs of diabetes distress*.

**Hypothesis** **2.** *We hypothesise that the Serbian version of the PAID-T will demonstrate concurrent validity with related measures, specifically showing positive correlations with HbA1c levels and negative correlations with Time in Range (TIR) and overall DES-28 scores. Furthermore, we anticipate gender differences in the perception of emotional burdens associated with diabetes management among adolescents; however, we do not expect significant differences across age groups within our sample*.

**Hypothesis** **3.** *We hypothesise that the Serbian version of the PAID-T will demonstrate convergent validity with other measures, specifically showing negative associations with social support from teachers, peers, and family members. In addition, we anticipate differences in how adolescents perceive the emotional burden of living with diabetes depending on their adherence to self-care activities, including glycaemic control, dietary regimen, physical activity, and diabetes management during school hours*.

**Hypothesis** **4.** *We hypothesise that the Serbian version of the PAID-T will demonstrate good reliability*.

## 2. Materials and Methods

### 2.1. Study Design

The study utilised a multicenter, cross-sectional, and validation research design. The translation and cultural adaptation of PAID-T and DES-28 into Serbian followed ISPOR guidelines [42] for the validation of patient-reported outcome measures. The STROBE Statement—Checklist for cross-sectional studies was used [43].

### 2.2. Setting and Participants

A comprehensive convenience sampling strategy was employed, resulting in a total of 374 adolescents with a confirmed diagnosis of T1D. Participants were categorised into two age groups representing both genders: younger adolescents (13–15 years) and older adolescents (16–18 years). This age-based stratification was selected to account for developmental differences, as older adolescents with T1D often encounter additional hormonal and psychosocial challenges that may heighten disease management difficulties.

Inclusion criteria: Adolescents were eligible for participation if they were between 13 and 18 years of age, had a confirmed diagnosis of T1D for at least three months, were proficient in reading and speaking Serbian, and did not present with cognitive impairments.

Exclusion criteria: Adolescents were excluded if they did not meet the above criteria. Specifically, this included individuals with diabetes types other than T1D, those unable to read or speak Serbian, and those with cognitive disabilities.

### 2.3. Sample Size Calculation

The required sample size was determined using the G*Power 3.1.9.7 [44] software for the following parameters:

- For the Wilcoxon rank-sum test, with an effect size (d) of 0.05, study power (1 − β) of 0.80, α error of 0.05, and an allocation ratio (n2/n1) of 9, the minimum sample size required was *n* = 368 (with 37 participants in group 1 and 331 in group 2).
- For linear multiple regression, assuming a small effect size (f^2^ = 0.03), a study power (1 − β) of 0.80, an α error of 0.05, and three predictors, the minimum sample size required was also *n* = 368.
- Our participant sample size, *n* = 374, was deemed sufficient for analysing the psychometric properties of the PAID-T. Recommendations for the ratio of items to respondents in validation studies vary from 1:10 to 1:5 [45]. Additionally, the minimum sample size for conducting CFA is recommended to be larger than 200 [46,47]. In our study, the total sample was divided into two independent subsamples, both of which were sufficiently large for factor analysis. The first subsample (*n* = 140) was used to perform EFA, allowing us to examine the underlying factor structure of the Serbian version of the PAID-T. The second subsample (*n* = 234) was then used to conduct CFA to validate the factor solution identified in the EFA. This procedure ensured that the EFA and CFA were performed on separate datasets, thereby minimising the risk of overfitting and increasing the robustness of our psychometric evaluation.

Note: given that the PAID-T had never been validated in Serbian or any other South Slavic language, we considered it essential to perform an EFA as a preliminary step to examine whether the underlying factor structure in our cultural and linguistic context aligned with that reported in international studies.

### 2.4. Survey and Data Collection

This study unfolded across three distinct stages:

1. The development of Serbian versions of the PAID-T and DES-28 was undertaken collaboratively across three institutions (details will be elaborated further in the manuscript).
2. Adolescents with T1D aged 13 to 18 (*n* = 374) who participated in this study attended routine check-ups or were hospitalised for various reasons, including transitioning from human insulin therapy to insulin analogues, receiving education on insulin pump usage, experiencing a deterioration in metabolic control, such as an episode of DKA, and undergoing re-education about diabetes management. All participants were in good physical and mental health at the time of the study. Adolescents with T1D were hospitalised in the Department of Endocrinology at two tertiary institutions: The Mother and Child Health Care Institute of Serbia “Dr. Vukan Čupić” and the University Children’s Hospital in Belgrade, as well as in the Department for treatment, education, and rehabilitation of children and youth at the Specialized “Bukovička Banja” Hospital in Arandjelovac, a secondary facility. Paediatric endocrinologists, co-authors of this report, and paediatric nurses referred adolescents with T1D and their parents/guardians to the principal investigator’s office.
3. Of the 374 adolescents with T1D enrolled in the study, *n* = 289 participants agreed to complete questionnaires in two stages for test–retest analysis: initially at the beginning of the study and again 10 to 15 days later, when the Serbian versions of the PAID-T and DES-28 were re-administered. Additionally, *n* = 85 adolescents consented to complete questionnaires during a single stage, either during routine check-ups or hospitalisation.

Note: A flow diagram of the study participants is shown in Figure S1 as Supplementary Material—File S1.

Ethical approval procedures required that each adolescent and/or their parent or guardian be provided with written information describing the study’s objectives and the exclusive scientific use of the data collected. Privacy and anonymity for adolescents with T1D were ensured, with only the authors having access to potentially identifying information during or after data collection.

Informed consent was obtained in writing, either from parents/guardians together with assent from children under 15, or from the patients themselves if they were 16 years or older, in line with the Serbian “Law on Patient Rights” (“Official Gazette of RS,” no. 45/2013 and 25/2019, Article 2, paragraphs 4–5) [48]. No additional interventions or diagnostic examinations were performed as part of the study. Participants maintained the right to decline specific questions or withdraw at any point, even after initial consent.

To ensure a conducive environment and privacy, adolescents completed surveys individually and without parental assistance in separate rooms, including sociodemographic data, adherence to self-care activities, and psychosocial instruments such as the PAID-T and DES-28 questionnaires. Throughout the process, parents or guardians were accommodated in a designated area, with a researcher available to address any queries or concerns. All questionnaires were administered concurrently, and incomplete responses were excluded from the analysis. The researcher meticulously recorded clinical information on a documentation sheet.

Data necessary to achieve the study’s objectives were collected in written form between 8 March 2022 and 29 April 2024.

### 2.5. Measures

The study questionnaire comprised five sections: (a) sociodemographic data, (b) clinical information, (c) a survey on adherence to self-care activities, and Serbian versions of (d) PAID-T and (e) DES-28 questionnaires.

#### 2.5.1. Sociodemographic Questionnaire

During the initial administration, all participants completed a sociodemographic questionnaire to gather details such as age (categorised as adolescents aged 13–15 and 16–18 years), gender, and self-reported assessments of social support from family, teachers, and peers. Social support was evaluated using a 4-point Likert scale (1 = no understanding/no support, to 4 = great understanding/great support), with an average score calculated across items. A score of 3 or higher indicated adequate social support, with higher scores indicating stronger perceived support.

Note: In the context of this research, social support was delineated as follows: “Social support refers to the perception and actuality that an individual is cared for and can receive assistance when needed from other people” [49].

#### 2.5.2. Clinical Data

Clinical data retrieved from the Heliant Health Information System electronic database included the duration of T1D (<5 or ≥5 years); glycosylated haemoglobin (HbA1c) levels categorised into ideal (<5.7%/<38.8 mmol/mol), good (5.7–6.9%/38.8–51.9 mmol/mol), unstable (7.0–8.5%/53.0–69.4 mmol/mol), and poor control (>8.5%/>69.4 mmol/mol); values of TIR; and presence of comorbidities.

#### 2.5.3. Assessment of Adherence to Self-Care Activities

Given the absence of Serbian assessment tools for evaluating self-care activities in adolescents with T1D, we employed a study-specific questionnaire, which was subjected to a two-step validation process. Content validity was assessed through expert review by one endocrinology faculty member and two paediatric nurses. In addition, face validity was established by obtaining feedback from adolescents aged 13–18 years, who assessed the clarity, simplicity, and comprehensibility of both the questions and the response options.

The assessment of adherence to self-care activities included items addressing glycaemic control, dietary practices, physical activity, and challenges encountered in diabetes management within the school setting.

Adherence to glycaemic control was determined based on the monitoring method employed (self-monitoring of blood glucose [SMBG] or CGM, the frequency of glucose checks, and either the maintenance of a blood glucose diary or the availability of current TIR values. Participants were categorised as either adherent or non-adherent. Patients were classified as adherent if they met one of the following criteria:

- SMBG group: Use of SMBG, achievement of recommended target glucose values (4.0–10.0 mmol/L [70–180 mg/dL], with a narrower fasting target of 4.0–8.0 mmol/L [70–144 mg/dL]), and reporting the response option indicating glucose monitoring at least three times daily before meals, along with postprandial checks (1.5–3 h after meals) at least three times per week [1,50].
- CGM group: Use of CGM with ≥70% of readings (over 14 days) within the recommended TIR of 3.9–10.0 mmol/L (70–180 mg/dL) [1,50].

Dietary adherence was assessed using medical records and closed-response queries to ensure they accurately represent the respondents’ answers and reduce bias risk.

Physical activity was evaluated based on engagement in daily moderate to vigorous physical activity and participation in sports for at least 60 min.

Challenges in diabetes management during school hours were assessed by inquiring about difficulties encountered (patients were categorised as adherent or non-adherent).

#### 2.5.4. Perceived Diabetes-Distress and Self-Efficacy

The Serbian versions of PAID-T and DES-28 questionnaires were utilised during initial and subsequent administrations. The translation and cultural adaptation of PAID-T and DES-28 into Serbian followed ISPOR guidelines [42].

Following initial translation (forward translation) by two independent translators and subsequent reconciliation, a pilot study with adolescents was conducted to ensure questionnaire clarity and comprehension. The translation of the Serbian version into English was performed independently by two other professional bilingual translators. Following the feedback, an expert panel reviewed both versions in Serbian and English. Minor changes were made, and the final version of the questionnaires was obtained.

#### 2.5.5. Perceived Diabetes-Distress

The permission to translate and use the 14-item PAID-T was obtained from the original developer [11], Jill Weissberg-Benchell, PhD, CDCES, Professor of Psychiatry and Behavioral Medicine (personal communication by email dated 10 July 2021). The 14-item PAID-T measures diabetes-specific distress in adolescents (12–18 years of age) with diabetes. Participants with diabetes rate how much each item bothered them over the past month using a 6-point Likert scale (1 = not a problem; 6 = serious problem), with higher scores indicating greater distress. The total PAID-T score can range from 14 to 84. Based on two observational studies, it was established that a cutoff score of 44 or higher is a significant measure of diabetes distress in adolescents. Moreover, Shapiro et al. [34] confirmed that PAID-T is a valid, reliable, and useful measure of diabetes-specific distress for teenagers, with an excellent Cronbach’s α of 0.93.

Note: The Serbian version of PAID-T was sent to Professor Jill Weissberg-Benchell.

#### 2.5.6. Perceived Self-Efficacy

Permission to translate and utilise the 28-item version of the Diabetes Empowerment Scale (DES), comprising three subscales—managing the psychosocial aspects of diabetes (nine items), assessing dissatisfaction and readiness to change (nine items), and setting and achieving diabetes goals (10 items)—was granted by the Michigan Diabetes Research Center (MDRC)/the Michigan Center for Diabetes Translational Research (MCDTR) with the decision “The project described was supported by Grant Number P30DK020572 (MDRC) from the National Institute of Diabetes and Digestive and Kidney Diseases”) (personal communication by email between the first author of the article and administrator PAM dated 23 July 2021). The scale uses a 5-point Likert format (1 = strongly disagree; 2 = disagree; 3 = neutral; 4 = agree; 5 = strongly agree). An overall score for the DES-28 is obtained by adding all the item scores and dividing the sum by 28. The higher the score, the higher the level of psychosocial self-efficacy [22,28]. The results from the Finnish study conducted on adolescents with T1D support the internal reliability of the DES-28, with a Cronbach’s alpha coefficient of 0.93 for the total scale and 0.75–0.89 for the subscales [24].

Note: The final Serbian version of DES-28 was subsequently used in the research and sent to the administrator PAM from MDRC/MCDTR.

Note: To ensure objectivity and minimise bias, data coding was conducted independently by three individuals. The verification of clinical information was carried out using multiple sources, including the Heliant Health Information System electronic database, medical records, and patients’ blood glucose diaries.

### 2.6. Data Analysis

Quantitative variables were summarised using standard descriptive statistics, including the arithmetic mean, standard deviation (SD), and median. To reduce the influence of extreme values, a trimmed mean was also calculated by removing the 5% of observations with the lowest and highest values. Variability was further described with the median absolute deviation (MAD), while the range was defined as the difference between the smallest and largest recorded values.

The shape of the data distribution was examined using skewness and kurtosis. Skewness reflects distribution asymmetry: negative values suggest that higher results dominate, whereas positive values indicate a predominance of lower results. Kurtosis provides information about the concentration of data around the mean relative to the tails: positive values imply a sharper peak with reduced spread, whereas negative values correspond to a flatter curve with greater dispersion. For data following a normal distribution, both skewness and kurtosis values are expected to approximate zero. The precision of the mean estimate was expressed through the standard error (SE). Categorical variables were summarised with frequencies (N) and percentages. The normality of distribution was assessed using the Shapiro–Wilk test for univariate data and the generalised Shapiro–Wilk test for multivariate data [51,52].

Construct Validity: The structure of the PAID-T questionnaire was evaluated through EFA and CFA [47,53].

Determining the Number of Factors: Various criteria, including parallel analysis [54], very simple structure [55], the Guttman–Kaiser criterion, the scree plot, and minimum average partial correlation [56], were employed to estimate the number of significant factors. Principal axis factoring was utilised as the extraction method, considering the data’s deviation from multivariate normality. The analysis was performed with 1000 iterations using the psych package [57].

Specification of Three Factors: For the three-factor models in CFA, parameters were estimated using the lavaan package [58] with a robust MLR estimator due to the non-normality of data. Models were identified by fixing the loading of the first item on each factor at 1. The semPlot package v.1.1.7 [59] was used for graphical presentation. Fit indices, including chi-square, comparative fit index (CFI), Tucker–Lewis Index (TLI), root mean square error of approximation (RMSEA), and standardised root mean square residual (SRMR), were evaluated based on Hu and Bentler’s recommendations. Acceptable fit statistics were defined as follows: a non-significant chi-square (*p* > 0.05) indicates a good fit. For an excellent fit, the CFI and TLI values needed to be ≥0.95, the RMSEA value required to be <0.05, and the SRMR value needed to be <0.08. For an acceptable fit, the required thresholds were CFI and TLI values ≥ 0.90, RMSEA < 0.08, and SRMR < 0.10 [60,61].

Reliability: Internal consistency was assessed using Cronbach’s alpha (α ≥ 0.70) [62] and McDonald’s omega coefficient (ω ≥ 0.70) [53]. Test–retest reliability was evaluated using the intraclass correlation coefficient (ICC) [57,63] for both average and absolute agreement, conducted over a retest period of 10 to 15 days. The recommended ICC value is ≥0.7 [63]. The semTools package v.0.4-14 [64] was used for reliability analysis on the sample of adolescents on whom CFA was performed.

Concurrent and Convergent Validity: The validity of the PAID-T scale was examined through correlations with relevant variables and differences between groups. Given that all variables deviate from the normal distribution (both in the sample as a whole and by groups), correlations were tested using Spearman’s rank correlation coefficient (ρ), and differences between groups by non-parametric substitutions for the *t*-test for independent samples and for one-way ANOVA (analysis of variance). Cohen’s Kappa was used to interpret the correlation level: insignificant (<0.1), low (0.1–0.3), moderate (0.3–0.5), and high (>0.5) [63,65,66]. The Wilcoxon signed-rank test, also known as the Mann–Whitney U test, was used to determine the differences between the two groups. The effect size in the above case was estimated using the Hedges’ g test, a variant of Cohen’s d. Hedges’ g is a corrected Cohen’s d that applies a correction for small samples because Cohen’s d is biased in small samples [67,68]. The rstatix package v.0.7.2 [69] facilitated these analyses.

Multiple Regression Analysis was performed to explore significant correlations between analysed variables and PAID-T scores (dependent variable).

All statistical analyses were conducted in the R programming language within the R Core Team’s Environment for Statistical Computing [69]. Data integrity was ensured by double-checking for entry errors and missing values before analysis. Results were presented in textual, tabular, or graphical formats, with statistical significance defined as *p* < 0.05.

## 3. Results

Among the 374 adolescents with T1D enrolled, 289 participants agreed to complete questionnaires in two stages: initially at the study’s outset and again 10–15 days later. Additionally, 85 adolescents consented to complete questionnaires in one stage.

### 3.1. Sociodemographic and Clinical Characteristics of Participants

Sociodemographic characteristics: A total of 374 adolescents diagnosed with T1D participated in the study, with 234 (63%) aged 13–15 years and 140 (37%) aged 16–18 years. The mean age of the participants was 14.95 years (SD = 1.59). Among the respondents, slightly more girls (190, 51%) than boys participated. The perception of social support was evaluated using mean scores: adolescents reported the lowest teacher support at 3.05 (SD = 0.34), peer support was estimated at 3.36 (SD = 0.32), and family support was the highest at 3.51 (SD = 0.57). The overall perception of social support had a mean score of 3.31 (SD = 0.27).

Clinical characteristics: Among the 374 adolescents with T1D, 190 (51.00%) had been managing the condition for five years or more, 97 (26%) had at least one comorbid condition, and the mean duration since T1D diagnosis was 5.99 years (SD = 3.91). The average HbA1c level was 8.14% (65.5 mmol/mol, SD = 1.67). Girls had a higher mean HbA1c level at 8.36% (67.90 mmol/mol, SD = 1.81) than boys at 7.91% (63.00 mmol/mol, SD = 1.49). Desired metabolic control (HbA1c < 7.0% or <53.00 mmol/mol) was observed in 87 adolescents (23.00%), while 166 (44%) had unstable control (HbA1c 7.0–8.5% or 53.0–69.4 mmol/mol), and 121 (32%) had poor control (HbA1c > 8.5% or >69.4 mmol/mol). CGM was utilised by 197 (52.67%) respondents, with occasional use reported by 17 (8.67%) adolescents, averaging 15.59 days per month (SD = 4.58). TIR data were available for 181 (48.40%) respondents, with 16 (8.12%) lacking TIR data.

### 3.2. Description of Adherence to Self-Care Activities

Non-adherence to glycaemic control was observed in 60 (16.04%) adolescents, while 179 (47.86%) did not adhere to dietary guidelines. Additionally, 273 (73.0%) were non-adherent to physical activity recommendations, and 278 (74.33%) reported difficulties managing T1D during school hours.

### 3.3. Descriptive Statistics for Psychosocial Characteristics of Adolescents with T1D

The mean PAID-T score was 43.79 (SD = 15.36) at the initial assessment and 43.52 (SD = 14.93) upon retest, with scores ranging from 14 to 84. The total DES-28 score averaged 2.34 (SD = 0.52) on a scale from 1 to 5. Among the subscales, the highest mean score was for managing the psychosocial aspects of diabetes (mean: 2.38, SD = 0.64), followed by setting and achieving diabetes goals (mean: 2.35, SD = 0.61) and assessing dissatisfaction and readiness to change (mean: 2.29, SD = 0.53).

### 3.4. Construct Validity of PAID-T: Exploratory and Confirmatory Factor Analysis

EFA was conducted on the first subsample (*n* = 140) to examine the underlying factor structure, followed by CFA on the second subsample (*n* = 234) to validate the structure of the Serbian adaptation of the PAID-T. All 374 adolescents (100%) included in the study completed the PAID-T in its entirety, with no missing responses.

#### 3.4.1. Determining the Number of Significant Factors

**Figure 1:**
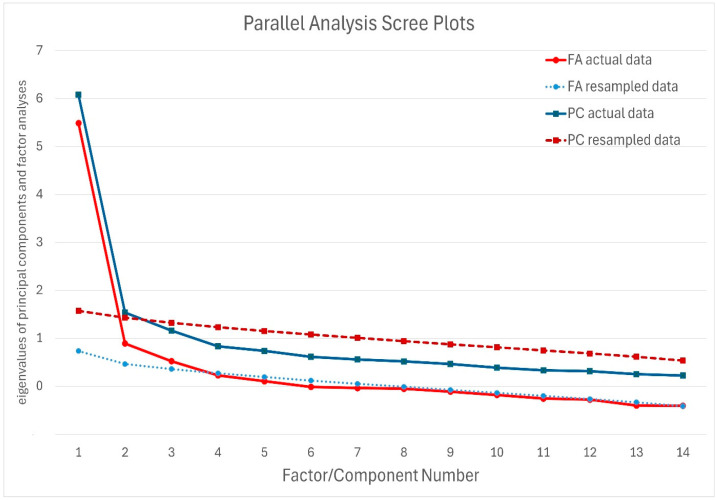
Parallel analysis.

Parallel analysis indicated three significant factors and two components (Figure 1).

Note: We selected the three-factor model based on previous research suggesting a prevalent three-factor solution with correlated factors.

#### 3.4.2. Exploratory Factor Analysis

EFA was conducted using the psych package. An analysis of the three-factor solution revealed factors representing emotional burden, distress among family and friends, and regimen-specific distress. The principal axis method was utilised for factor extraction, and factors were rotated using the promax rotation method.

Together, these factors accounted for 46.5% of the shared variance. The first factor accounted for the largest portion at 22%, followed by the second at 15.1%, and the third at 9.4% (refer to Table 1).

Correlations between the second and third factors and the first were notably high (0.62 each), while their correlation with each other was 0.43, indicating a moderate to high relationship (as shown in Table 2).

This suggests that these three distinct factors likely represent facets or subdomains of the same underlying construct.

#### 3.4.3. Confirmatory Factor Analysis

CFA examined multiple models to describe the data structure. Initially, a single-factor model was tested based on previous research in other languages and indications from several EFA criteria, which suggested a potential single-factor solution. Subsequently, a three-factor model with correlated factors and a hierarchical model featuring three first-order factors and one second-order factor were evaluated. Additionally, a bifactor model incorporating three specific factors corresponding to those identified in the three-factor solution was assessed; however, this model was rejected as misspecified because it led to negative variances for individual items.

**Figure 2:**
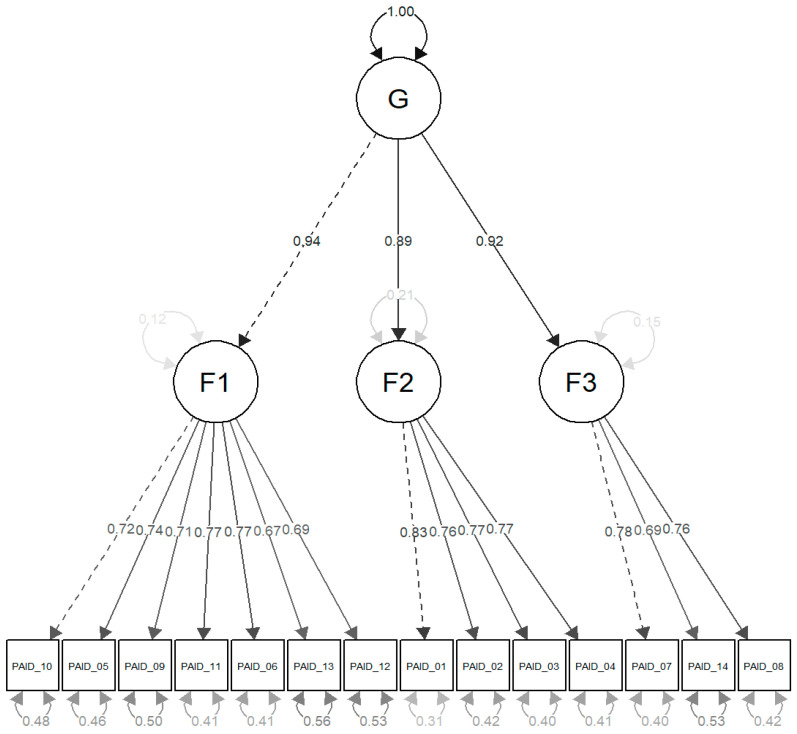
Hierarchical three-factor model. Legend: G—one general factor; F1—the first factor (regimen-specific distress) included items 10, 5, 9, 11, 6, 13, and 12; F2—the second factor (emotional burden) comprised items 1, 2, 3, and 4; and F3—the third factor (family and friends distress) encompassed items 7, 14, and 8. Solid paths represent freely estimated parameters, whereas dashed paths denote marker items whose loadings were fixed at 1 in order to achieve model identification. The figure was created using the semPlot R-package [59].

To specify the three factors in the three-factor models, findings from EFA guided the assignment of items: the first factor (regimen-specific distress) included items 10, 5, 9, 11, 6, 13, and 12; the second factor (emotional burden) comprised items 1, 2, 3, and 4; and the third factor (family and friends distress) encompassed items 7, 14, and 8 (see Figure 2).

These models were estimated using the lavaan package with the robust MLR estimator, chosen to accommodate data non-normality. Model identification was achieved by fixing the loading of the first item on each factor at 1.

Chi-square, absolute fit indices, and approximate fit indices such as the comparative fit index (CFI), Tucker–Lewis Index (TLI), root mean square error of approximation (RMSEA), and standardised root mean square residual (SRMR) were utilised as indicators of model fit. CFI and TLI values needed to be ≥0.95, RMSEA < 0.05, and SRMR < 0.08 to qualify as an excellent fit. For an acceptable fit, CFI and TLI values were required to be ≥0.90, RMSEA < 0.08, and SRMR < 0.10. The three-factor model with correlated factors and the hierarchical three-factor model (Figure 1) demonstrated identical and satisfactory fit indicators. Therefore, despite some multidimensionality, the PAID-T scale can be considered a measure of a single construct, allowing for the calculation of a total score.

Note: The bi-factor model, which incorporated three specific factors, showed the best-fit indicators; however, it presented negative variances for some items, indicating misspecification. The single-factor model did not achieve an acceptable fit. The fit statistics for the CFA models of the PAID-T are presented in Table 3.

Table 4 presents a description of the factor loadings of items on the first-order factors.

Table 5 presents a description of the factor loadings of the first-order factors on the second-order factors.

### 3.5. Reliability: PAID-T and DES-28

#### 3.5.1. Reliability: PAID-T

The reliability of the Serbian PAID-T scale was confirmed using a hierarchical three-factor model (α_F1_ = 0.883; α_F2_ = 0.864; α_F3_ = 0.784; and ω_F1_ = 0.884; ω_F2_ = 0.865; ω_F3_ = 0.786). The reliability of the total score (with all 14 items) was determined by the high Cronbach’s alpha coefficient (α = 0.92), but also by applying the one-factor model with a high McDonald’s omega coefficient (ω = 0.93). The Serbian version exhibited strong test–retest reliability (both on total score and three subscales), with an ICC of 0.99 based on the 95% confidence interval, indicating excellent reproducibility. Adolescents (*n* = 289) completed the questionnaire at intervals of 10 to 15 days to minimise recall bias.

#### 3.5.2. Reliability: DES-28

Regarding the Serbian version of the DES-28 questionnaire, internal consistency was also strong, as indicated by a Cronbach’s alpha coefficient of 0.93 and a McDonald’s omega coefficient of 0.95.

### 3.6. Concurrent Validity: PAID-T

Based on previous research findings, the concurrent validity was investigated by correlating the Serbian version of PAID-T (both test and retest) with clinical variables (HbA1c and TIR). The analysis revealed no significant associations between PAID-T scores and HbA1c levels or TIR (refer to Table 6).

Additionally, correlations were examined between the Serbian versions of DES-28 and PAID-14 (both test and retest). The results indicated a positive correlation between the overall scores of the DES-28 and PAID-14 (ρ = 0.32, *p* = 0.00, with multiple analysis correction). Further detailed statistical information regarding the analysed correlations can be found in Table 6.

Additionally, as anticipated, differences were observed in how adolescents perceive the emotional burden of living with diabetes based on their gender. Female adolescents reported significantly higher diabetes-specific distress (median = 46.00) than male adolescents (median = 42.5), with a Wilcoxon test statistic of W = 19,718.0, *p* = 0.03, indicating a small effect size of 0.11. Furthermore, there were no significant differences in emotional burden perception across different age groups for both the initial assessment (W = 15,972.0, *p* = 0.82, small effect size = 0.01) and retest (W = 9829.0, *p* = 0.96, small effect size = 0.00).

As expected, a significant negative correlation was found between PAID-T scores and total social support scores (ρ = −0.24, *p* = 0.00 for the test; ρ = −0.25, *p* = 0.00 for the retest), indicating that higher distress was associated with lower perceived social support. Detailed statistical results of these correlations are provided in Table 6.

Moreover, differences in perceived emotional burden were noted regarding adherence to self-care activities. Specifically, adolescents who were non-adherent to glycaemic control reported significantly higher diabetes-specific distress (median_test_ = 50.00) compared to those who adhered to glycaemic control (median_test_ = 44.00), with a Wilcoxon test statistic of W = 11,390.0, *p* = 0.01, and a small effect size of 0.13 for the initial assessment. This finding was confirmed upon retesting (median_retest_ = 50.00 vs. median_retest_ = 43.50, W = 7068.0, *p* = 0.02, small effect size = 0.13).

Similarly, adolescents who faced challenges managing diabetes at school reported higher diabetes-specific distress (median_test_ = 46.00) compared to those without such difficulties (median_test_ = 37.00), with a Wilcoxon test statistic of W = 16,333.5, *p* = 0.00, and a small effect size of 0.16 for the initial assessment. This result was also confirmed upon retesting (median_retest_ = 47.00 vs. median_retest_ = 36.00, W = 10,127.0, *p* = 0.00, small effect size = 0.19). Other examined differences did not reach statistical significance.

### 3.7. Multiple Regression Analysis

A multivariate linear regression model was constructed to explore whether adherence to glycaemic control (adherent vs. non-adherent), the management of T1D during school hours (difficulties vs. no difficulties), and self-reported social support (overall score) predict diabetes distress in adolescents with T1D (dependent variable).

The analysis revealed that managing T1D during school hours negatively predicted diabetes distress (β = −0.28, t = −2.39, *p* = 0.01). This suggests that adolescents who reported no difficulties in managing their condition at school had lower scores on the PAID-T compared to those facing challenges. Higher self-assessed social support also negatively predicted diabetes distress (β = −0.22, t = −4.32, *p* = 0.00). The standardised coefficients (β) indicate that the impact of managing T1D during school hours appears slightly stronger than that of social support, although this difference was not statistically significant (Z = −0.178).

Notably, there was no significant effect of adherence to glycaemic control on diabetes distress (see Table 7 and Table 8). However, according to the Wilcoxon test, non-adherent adolescents did show differences in PAID-T scores.

## 4. Discussion

This research aimed to develop a valid and reliable Serbian version of the PAID-T through meticulous translation and adaptation. The findings from testing the factor structure and psychometric properties of the Serbian 14-item PAID-T were promising, confirming most of our hypotheses. Consequently, this study marks the first report on diabetes-specific distress among Serbian adolescents with T1D.

The first hypothesis was confirmed. Construct validity was assessed using EFA and CFA to validate the PAID-T structure. To strengthen the methodological rigour of our study, we deliberately divided the total sample into two independent subsamples, performing EFA on the first subsample (*n* = 140) and CFA on the second (*n* = 234). This approach ensured that the exploratory and confirmatory analyses were not conducted on the same participants, thereby minimising the risk of model overfitting. Such a procedure is consistent with best practices recommended in psychometric and cross-cultural validation studies, where independent samples are advised to establish both the stability and generalizability of the factor structure across different contexts. Our results indicated that both the three-factor correlated model and the hierarchical three-factor model with one second-order factor have acceptable and equal fit indicators. However, despite certain multidimensionality, the high correlations between factors in the three-factor model and the best indicators of fit for the hierarchical three-factor model with one second-order factor indicate that the PAID-T has a unified construct of measurement, which is consistent with conclusions drawn by Shapiro et al. (at the original version) [34]. Saßmann et al. [37] similarly identified three core constructs, namely emotional burden, distress among family and friends, and regimen-specific distress, which collectively accounted for the total score. This validated Serbian version of PAID-T will aid multidisciplinary teams caring for adolescents with T1D in Serbia. Diabetes distress is linked to clinical outcomes and psychosocial well-being. Diabetes distress is predictive of unsatisfactory metabolic control, worse treatment satisfaction, and poor adherence to self-care activities, which, in turn, can increase the risk of DKA. In addition, the negative impact of diabetes distress is evident in the entire social functioning. Therefore, clinicians could quickly review adolescents’ performance on the PAID-T and discuss responses during clinical reviews.

The second hypothesis was partially confirmed. More precisely, concurrent validity was explored by correlating PAID-T with clinical variables (HbA1c and TIR) and DES-28, a tool for assessing self-efficacy in T1D patients. Unexpectedly, no significant associations were found between the Serbian PAID-T (both test and retest) and HbA1c levels or TIR, contradicting findings from Hagger et al. [8] and Saßmann et al. [37]. These results may reflect socially acceptable responses or the impact of coping with puberty. Therefore, paediatric nurses should specifically engage with adolescents who have poor metabolic control, emphasising the importance of achieving an HbA1c level < 7.0% (53.0 mmol/mol) and maintaining an optimal TIR above 70% at least 17 h (more precisely >16 h 48 min) of a 24 h day. This is crucial for slowing the progression of both microvascular and macrovascular complications [37]. Notably, we observed an unexpected positive correlation between higher self-efficacy and increased diabetes distress, which contrasts with findings in adults [31]. This divergence may stem from differences in patient populations (adolescents vs. adults), their distinct psychosocial and developmental stages, and varied life experiences and challenges. It may also suggest that Serbian adolescents who are more aware of their diabetes-related issues perceive themselves as having higher self-efficacy. These results may be a consequence of adolescents striving for independence while managing their condition [1,18]. Previous research highlighted that self-empowerment is a gradual process with many peaks and troughs. Since adolescents with T1D are navigating various psychological and physiological changes [70], Anderson et al. [30] discuss how self-efficacy can develop through an empowerment approach, emphasising its role in coping with T1D. Health professionals and caregivers should empower adolescents with T1D to manage their self-care activities while providing appropriate support. Nonetheless, it can be argued that the second hypothesis is partially supported, as no significant age-related differences were found in the perception of diabetes-related emotional burden, which is consistent with the findings of Saßmann et al. [37]. Furthermore, female adolescents reported significantly higher diabetes-specific distress, aligning with prior research [8,13,36,37,71], thereby validating the criterion validity of the Serbian PAID-T version. Clinicians must be aware of the adolescent’s evolving developmental stages and adapt holistic care to meet the individual needs and circumstances of these paediatric patients.

The third hypothesis was mostly confirmed. More precisely, convergent validity was assessed through correlations between the Serbian PAID-T and social support from teachers, peers, and family, as well as differences in how adolescents perceive the emotional burden of living with diabetes depending on their adherence to self-care activities, including glycaemic control, dietary regimen, physical activity, and diabetes management during school hours. Statistically significant differences were observed in perceiving the diabetes burden in terms of overall social support and family support, echoing findings from Weissberg-Benchell and Antisdel-Lomaglio [13], which confirms the third hypothesis. Adequate social support has been shown to mitigate distress and promote resilience among adolescents with T1D [72,73]. However, peer support did not reach statistical significance in our study, consistent with Luo et al.’s findings [74], although peers play a crucial role in adolescents’ emotional well-being and self-esteem [75].

Moreover, managing T1D during school hours emerged as a significant factor influencing diabetes distress, aligning with the findings of Luo et al. [41] and Nannsen et al. [76] and confirming the third hypothesis. Adolescents facing difficulties in school-related diabetes management reported higher distress levels compared to those without challenges, underscoring the need for a supportive and adaptable school environment. Similar challenges were noted among Serbian paediatric patients with T1D in previous studies [1,77]. Implementing ISPAD guidelines for diabetes management in schools is crucial, emphasising the importance of trained school personnel to support diabetes care throughout the day [78]. However, previous studies suggest that teachers’ roles in diabetes management should be further explored to enhance educational interventions and support strategies [79,80].

Furthermore, non-adherence to glycaemic control was associated with greater diabetes distress, consistent with previous research [6,11,32,35], which affirms the convergent validity of the Serbian PAID-T version. Therefore, structured diabetes education and re-education should include problem-solving support and stress management strategies to help reduce diabetes distress among adolescents with T1D, particularly for female adolescents. Adherence to a nutritious diet and regular physical activity are key components of diabetes self-management. However, current evidence is limited in exploring how adolescents perceive the emotional burdens of living with diabetes in relation to their adherence to dietary regimens and physical activity.

Our findings suggest that there are no significant differences in how adolescents perceive diabetes distress based on their level of physical activity. This contrasts with the findings of Tilden et al. [81], who reported that moderate-to-vigorous physical activity was associated with lower diabetes distress among adolescents with T1D. Similarly, we found no significant differences in diabetes distress based on adherence to dietary guidelines, which contrasts with the findings of Araia et al. [82], who reported a positive association between diabetes distress and disordered eating in adolescents with T1D.

Thus, our findings do not fully support the third hypothesis. These discrepancies may be explained by variations in coping strategies and differences in diabetes management. Specifically, there may be instances where adolescents are less willing to acknowledge the emotional burdens of living with diabetes and may cope through different behaviours or actions.

The fourth hypothesis was confirmed. The reliability: The PAID-T questionnaire demonstrated strong internal consistency, with Cronbach’s alpha coefficient measuring 0.92 and McDonald’s omega coefficient measuring 0.93, which is similar to other studies [13,34,37]. The test–retest intraclass correlation coefficient (ICC) was excellent, with a value of 0.99, as indicated by the 95% confidence interval. Adolescents (*n* = 289) completed the questionnaire at intervals of 10 to 15 days to minimise recall bias.

In conclusion, Serbian adolescents with T1D reported PAID-T scores close to the cutoff point of 44, which is significantly higher than those observed in German adolescents (43.79 for the Serbian-PAID-T test and 43.52 for the Serbian-PAID-T retest, compared to 33.98 for the German-PAID-T). Additionally, adherence to self-care activities in Serbian children and adolescents with T1D remains suboptimal, consistent with recent investigations [1]. These findings underscore the ongoing challenges in maintaining consistent self-care routines among adolescents with T1D [15,16,76,81,82].

Moreover, the multivariate linear regression model confirmed that understanding school-related diabetes management challenges and evaluating social support are crucial in predicting diabetes-specific distress among adolescents with T1D. These results align with Markowitz et al.’s [83] review, which emphasises the importance of supportive environments for adolescents with T1D, as well as the gradual transition of diabetes management responsibilities from parents to adolescents, known to influence both physiological and psychosocial outcomes. The ISPAD guidelines recommend routine screening for diabetes distress in adolescents aged 12 and older, using validated tools, particularly during transitions in disease management or life circumstances [84].

### 4.1. Perspectives for Clinical Practice

Despite ongoing efforts by healthcare teams—including treatment, care, education, re-education, and psychosocial support for paediatric patients with T1D—suboptimal metabolic control, issues related to inadequate self-efficacy, and pronounced emotional distress persist among adolescents. These challenges remain prevalent not only in the Republic of Serbia but also globally, despite advancements in evidence-based medicine and increased accessibility to modern medical technologies for diabetes management. Consequently, identifying the underlying causes of poor health outcomes—both clinical and humanistic—in adolescent populations with T1D necessitates a multidisciplinary approach. This approach should involve collaboration among healthcare professionals across all levels of care, such as paediatric endocrinologists, paediatric nurses specialised in diabetes education, psychologists, dietitians, ophthalmologists, neurologists, nephrologists, social workers, and primary care paediatricians, with active participation from parents or guardians.

Drawing on research findings and the reviewed literature, various interventions should be adopted to enhance adolescents’ capacity for self-management of T1D. These include the following:

(1) Augmenting the formal education of primary healthcare providers—particularly paediatricians and paediatric nurses—with additional training focused on the recognition of diabetes-related emotional distress and current guidelines for optimal disease control and self-management.
(2) Implementing screening procedures to identify diabetes-specific emotional distress, thereby safeguarding and promoting adolescents’ mental health.
(3) Introducing assessments to evaluate adolescents’ subjective beliefs regarding their self-management capabilities within various life contexts, including family and school environments, as well as their readiness for transitioning from paediatric to adult healthcare services.

### 4.2. Strengths and Weaknesses

The primary strength of our study lies in the use of two validated and reliable standardised instruments (PAID-T and DES-28) to assess psychosocial outcomes in adolescents with T1D. Additionally, our research included a representative sample of adolescents with T1D from all regions of Serbia, including Belgrade, Vojvodina, southern and eastern Serbia, Sumadija, and western Serbia.

However, the possible generalisation of the collected data, the type of study conducted, and the tool adopted must be considered. Therefore, the results should be interpreted with several limitations in mind. Firstly, due to the lack of translated and validated questionnaires in Serbian that address adherence to self-care activities and social support for adolescents with T1D, the self-report questionnaires used in this study were developed by the lead researcher and co-authors. Secondly, the reliance on self-reported data across the three questionnaires introduces potential social and recall biases. Additionally, important data on socioeconomic status, a significant factor in adherence to self-care activities and a mediator of psychosocial well-being, were not collected during the enrolment process. Future research could incorporate longitudinal data collection to examine the predictive validity of the Serbian PAID-T over time, particularly regarding its association with long-term glycaemic control and psychosocial outcomes.

## 5. Conclusions

The Serbian version of the PAID-T demonstrated strong validity and reliability in assessing diabetes-specific distress among adolescents aged 13–18 years with T1D. Factor analyses supported treating the PAID-T as a measure of a unified construct, as evidenced by the calculation of the total score, despite some evidence of multidimensionality. Importantly, Serbian adolescents reported mean PAID-T scores close to the established cutoff point of 44, indicating clinically relevant levels of diabetes-specific distress within this population.

Our findings further revealed that challenges in managing diabetes at school, adherence to glycaemic control, and the adequacy of social support significantly influenced reported distress levels. Specifically, the multivariate linear regression model confirmed that school-related difficulties and lower perceived social support were pivotal predictors of diabetes-specific distress. These results highlight the need to move beyond an individual-centred approach and consider both contextual and interpersonal factors when addressing the psychosocial burden of diabetes.

From a clinical perspective, the PAID-T should be strongly advocated as a valuable tool for the early detection and systematic evaluation of diabetes distress in both research and routine paediatric diabetes care. Consistent with ISPAD guidelines [84], incorporating such validated tools into routine screening can enable nurses and other members of the multidisciplinary healthcare team to identify adolescents at higher risk, provide targeted psychosocial support, and ultimately improve both psychological well-being and metabolic outcomes.

At the same time, these results underscore the broader implications for healthcare systems and public health. Cultural adaptation and validation of the PAID-T ensures its conceptual pertinence within local healthcare contexts, while the identification of distress close to clinically significant thresholds points to an urgent need for targeted interventions. A particularly pressing issue in countries with underdeveloped healthcare and social protection systems is the unequal access to modern diabetes self-management technologies. Addressing these disparities is essential to ensure equity in care and to reduce the psychosocial burden faced by adolescents with T1D. Furthermore, the advancement of precision medicine in type 1 diabetes offers promising opportunities to tailor interventions to individual patient profiles, combining medical, technological, and psychosocial strategies for optimal outcomes.

## Figures and Tables

**Table 1:** Factors analysis.

Item	F1	F2	F3
PAID_10	0.84		
PAID_05	0.81		
PAID_09	0.80		
PAID_11	0.64		
PAID_06	0.61	0.32	
PAID_13	0.46	0.30	
PAID_12	0.41		
PAID_01		0.87	
PAID_02		0.76	
PAID_03		0.75	
PAID_04		0.45	
PAID_07			0.89
PAID_14			0.59
PAID_08			0.46

Legend: F1—the first factor (regimen-specific distress); F2—the second factor (emotional burden); F3—the third factor (family and friends distress).

**Table 2:** Factor correlations.

Factor	F1	F2	F3
F1	1.00	0.62	0.62
F2	0.62	1.00	0.43
F3	0.62	0.43	1.00

Legend: F1—the first factor (regimen-specific distress); F2—the second factor (emotional burden); F3—the third factor (family and friends distress).

**Table 3:** Fit statistics for CFA models of the PAID-T.

Model	χ^2^	Df	*P*	CFI	TLI	RMSEA	Lower	Upper	RMSEA *p*	SRMR
Single-factor model	205.17	77.00	0.00	0.90	0.88	0.10	0.08	0.12	0.00	0.06
Three-factor model	141.09	74.00	0.00	0.95	0.93	0.08	0.06	0.09	0.02	0.05
Hierarchical three-factor model	141.09	74.00	0.00	0.95	0.93	0.08	0.06	0.09	0.02	0.05
Bi-factor model	104.75	63.00	0.00	0.97	0.95	0.06	0.04	0.08	0.16	0.04

Legend: Chi-square test (χ^2^), degrees of freedom (df); *p*-value; comparative fit index (CFI); Tucker–Lewis index (TLI); root mean square error of approximation (RMSEA); standardised root mean square residual (SRMR). Note: A significant χ^2^ test does not necessarily suggest poor model fit as it is considered highly sensitive in large samples [54,60,61].

**Table 4:** Factor loadings of items to first-order factors.

Item	F1	F2	F3	Se	*P*
PAID_10	0.72			0.05	0.00
PAID_05	0.74			0.04	0.00
PAID_09	0.71			0.04	0.00
PAID_11	0.77			0.03	0.00
PAID_06	0.77			0.03	0.00
PAID_13	0.67			0.04	0.00
PAID_12	0.69			0.04	0.00
PAID_01		0.83		0.03	0.00
PAID_02		0.76		0.04	0.00
PAID_03		0.77		0.03	0.00
PAID_04		0.77		0.04	0.00
PAID_07			0.78	0.04	0.00
PAID_14			0.69	0.05	0.00
PAID_08			0.76	0.04	0.00

Legend: F1—the first factor (regimen-specific distress); F2—the second factor (emotional burden); F3—the third factor (family and friends distress); se—denotes the standard error; *p*-values.

**Table 5:** Factor loadings of first-order factors to the second-order factor.

Factor	G	Se	*P*
F1	0.94	0.03	0.00
F2	0.89	0.03	0.00
F3	0.92	0.04	0.00

Legend: F1—the first factor (regimen-specific distress); F2—the second factor (emotional burden); F3—the third factor (family and friends distress); G—second-order factor; se—denotes the standard error; *p*-values.

**Table 6:** Spearman’s rank correlation.

ρ	TIR	Social Support (Teachers)	Social Support (Peers)	Social Support (Family)	Social Support (Total Score)	HbA1c %	PAID-T (Test)	PAID-T (Retest)	DES-28 (Total Score)	DES MPSAD	DES ADaRC	DES SaADG
TIR		0.11	0.05	−0.01	0.06	−0.62 **	−0.06	−0.02	−0.32 **	−0.30 **	−0.29 *	−0.25
Social support (teachers)			0.22 **	0.10	0.58 **	−0.03	−0.13	−0.14	−0.09	−0.09	−0.05	−0.10
Social support (peers)				0.12	0.55 **	−0.12	−0.04	−0.06	−0.15	−0.12	−0.07	−0.19 *
Social support (family)					0.78 **	−0.16	−0.23 **	−0.22 *	−0.29 **	−0.24 **	−0.22 **	−0.31 **
Social support (total score)						−0.14	−0.24 **	−0.25 **	−0.29 **	−0.24 **	−0.19 *	−0.32 **
HbA1c %							0.09	0.06	0.43 **	0.45 **	0.35 **	0.34 **
PAID-T (test)								0.99 **	0.32 **	0.18 *	0.23 **	0.44 **
PAID-T (retest)									0.32 **	0.16	0.23 *	0.44 **
DES-28 (total score)										0.91 **	0.88 **	0.84 **
DES MPSAD											0.75 **	0.64 **
DES ADaRC												0.59 **
DES SaADG												

Legend: ρ coefficient: Significant ρ coefficients with multiple-analysis correction and *p*-values ** < 0.01 and * < 0.05 are shown in bold. Glycosylated haemoglobin (HbA1c); time in range (TIR) 3.90–10.00 mmol/L (70–180 mg/dL) for the last 14 days; Problem Areas in Diabetes scale—teen version (PAID-T); Diabetes Empowerment Scale (DES); Managing the Psychosocial Aspects of Diabetes (MPSAD); Assessing Dissatisfaction and Readiness to Change (ADaRC); Setting and Achieving Diabetes Goals (SaADG).

**Table 7:** Multivariate regression model on the effects of adherence to glycaemic control, self-control of T1D during school stay, and social support on diabetes distress.

	Β	se_B_	T	*p*
(Intercept)	0.024	0.069	0.348	0.728
Adherence to glycaemic control (Adherent)	−0.034	0.117	−0.293	0.770
Self-control of T1D during school stay (No difficulties in school)	−0.287	0.120	−2.391	0.017
Social support (Overall score)	−0.239	0.055	−4.326	0.000

Note: Since the PAID-T score does not have a normal distribution and neither does social support, these two variables were previously normalised. (Based on the percentile rank of each score on each of these two variables, z-values that correspond to those percentile scores in a normal distribution were found, so they were not simply converted into z-scores based on the standard deviation.) A linear model was estimated using the ordinary least squares (OLS) method. The model explains a statistically significant but small proportion of the dependent variable (R^2^ = 0.07, F(3, 369) = 9.88, *p* < 0.001, adjusted R^2^ = 0.07). The 95% CI and *p*-values were calculated using the Wald approximation of the t-distribution.

**Table 8:** Standardised coefficients.

	β	95% CI
Adherence to glycaemic control (Adherent)	−0.03	−0.26 −0.19
Self-control of T1D during school (No difficulties in school)	−0.28	−0.51 −0.05
Social support (Overall score)	−0.22	−0.32 −0.12

## Data Availability

The data supporting the findings of this study are available on request from the corresponding author (M.S.).

## Supplementary Materials

The following supporting information can be downloaded at: https://www.mdpi.com/article/10.3390/nursrep15090326/s1, Supplementary File S1. Figure S1. A flow diagram of the study participants. Supplementary File S2. PAID-T_questionnaire_backward translation_ENG_validation_SRB. Supplementary File S3. STROBE checklist.

## Public Involvement Statement

No public involvement in any aspect of this research.

## Guidelines and Standards Statement

This manuscript was drafted against the STROBE Statement—Checklist for cross-sectional studies. The translation and cultural adaptation of PAID-T and DES-28 into Serbian were conducted in accordance with ISPOR guidelines.

## Abbreviations

The following abbreviations are used in this manuscript:

β	The standardised beta coefficients
ADaRC	Assessing dissatisfaction and readiness to change
CFA	Confirmatory factor analysis
CFI	Comparative fit index
CGM	Continuous glucose monitoring
CI	Confidence interval
DES	Diabetes empowerment scale/score
df	Degrees of freedom
DSNs	Diabetes specialist nurses
G	One general factor
GAD-65	Glutamic acid decarboxylase-65
EFA	Exploratory factor analysis
EMA	European Medicine Agency
HbA1c	Glycosylated haemoglobin
IAA	Insulin autoantibodies
IA-2A	Insulinoma-associated-2 autoantibodies
ICC	Intraclass correlation coefficients
ISPAD	International Society for Pediatric and Adolescent Diabetes
Mad	Median absolute deviation
MDRC	Michigan Diabetes Research Center
MCDTR	Michigan Center for Diabetes Translational Research
MPSAD	Managing the psychosocial aspects of diabetes
OLS	Ordinary least squares
PAID	Problem areas in diabetes
RMSEA	Root mean square error of approximation
SaADG	Setting and achieving diabetes goals
SD	Standard deviation
SMBG	Self-monitoring of blood glucose
SRMR	Standardised root mean square residual
STROBE	The Strengthening the Reporting of Observational Studies in Epidemiology
ρ	Spearman’s rank correlation coefficient
T1D	Type 1 diabetes
TIR	Time in range
TLI	Tucker–Lewis index
W	Wilcoxon rank-sum test
Χ^2^	Chi-square test
ZnT8	Zinc transporter 8 (ZnT8)
