# Supplementary material for: Psychometric Properties of the Serbian Teen Version of the Problem Areas in Diabetes Scale—A Validation Study
Source: Nurs Rep. 2025 Sep 8;15(9):326. doi: 10.3390/nursrep15090326 (PMC12472196; doi:10.3390/nursrep15090326)
Supplement: Supplementary file 1 [file nursrep-15-00326-s001.zip › Supplementary File S2. PAID-T backward translation_ENG.pdf]

IDENTIFYING YOUR PROBLEM AREAS IN DIABETES − TEEN VERSION (PAID-T)

Name: ________________________________ Age: _____ Sex: M__ F __
How old were you when your diabetes was diagnosed? ______
Today's date: ______________

DIRECTIONS: Living with diabetes can sometimes be difficult. In everyday life, many problems and hassles with your diabetes can emerge. These problems may range from minor issues to major life difficulties. Listed below are diverse potential problem areas that people with diabetes may have. Think about how much each of the items below may have upset or bothered you **DURING THE PAST MONTH** and circle the appropriate number.

Please note that we are asking you how much each item may be bothering you in your life, **NOT** whether the item is merely true for you. If you feel that an item is not an issue or a problem for you, you should circle "1". If it is very bothersome to you, you should circle "6".

	Not a Problem		Moderate Problem		Serious Problem	
1. Feeling sad when I think about having and living with diabetes.	1	2	3	4	5	6
2. Feeling overwhelmed by my diabetes regimen.	1	2	3	4	5	6
3. Feeling angry when I think about having and living with diabetes.	1	2	3	4	5	6
4. Feeling "burned out" by the constant effort to manage diabetes.	1	2	3	4	5	6
5. Feeling that I am not checking my blood sugars often enough.	1	2	3	4	5	6
6. Not feeling motivated to keep up with my daily diabetes tasks.	1	2	3	4	5	6
7. Feeling that my friends or family act like "diabetes police" (e.g., nag me about eating properly, checking blood sugars, not trying hard enough).	1	2	3	4	5	6
8. Feeling that my parents don't trust me to care for my diabetes.	1	2	3	4	5	6
9. Missing or skipping blood sugar checks.	1	2	3	4	5	6
10. Feeling that I am often failing with my therapeutic regimen of diabetes.	1	2	3	4	5	6

11. Feeling like my parents blame me for blood sugar values they don't like.	1	2	3	4	5	6
12. Feeling that my friends or family don't understand how difficult living with diabetes can be.	1	2	3	4	5	6
13. Worrying that diabetes is preventing me from having fun and being with my friends.	1	2	3	4	5	6
14. Feeling like my parents worry about complications too much.	1	2	3	4	5	6
