# Supplementary figures and images for: Psychometric Properties of the Serbian Teen Version of the Problem Areas in Diabetes Scale—A Validation Study
Source: Nurs Rep. 2025 Sep 8;15(9):326. doi: 10.3390/nursrep15090326 (PMC12472196; doi:10.3390/nursrep15090326)

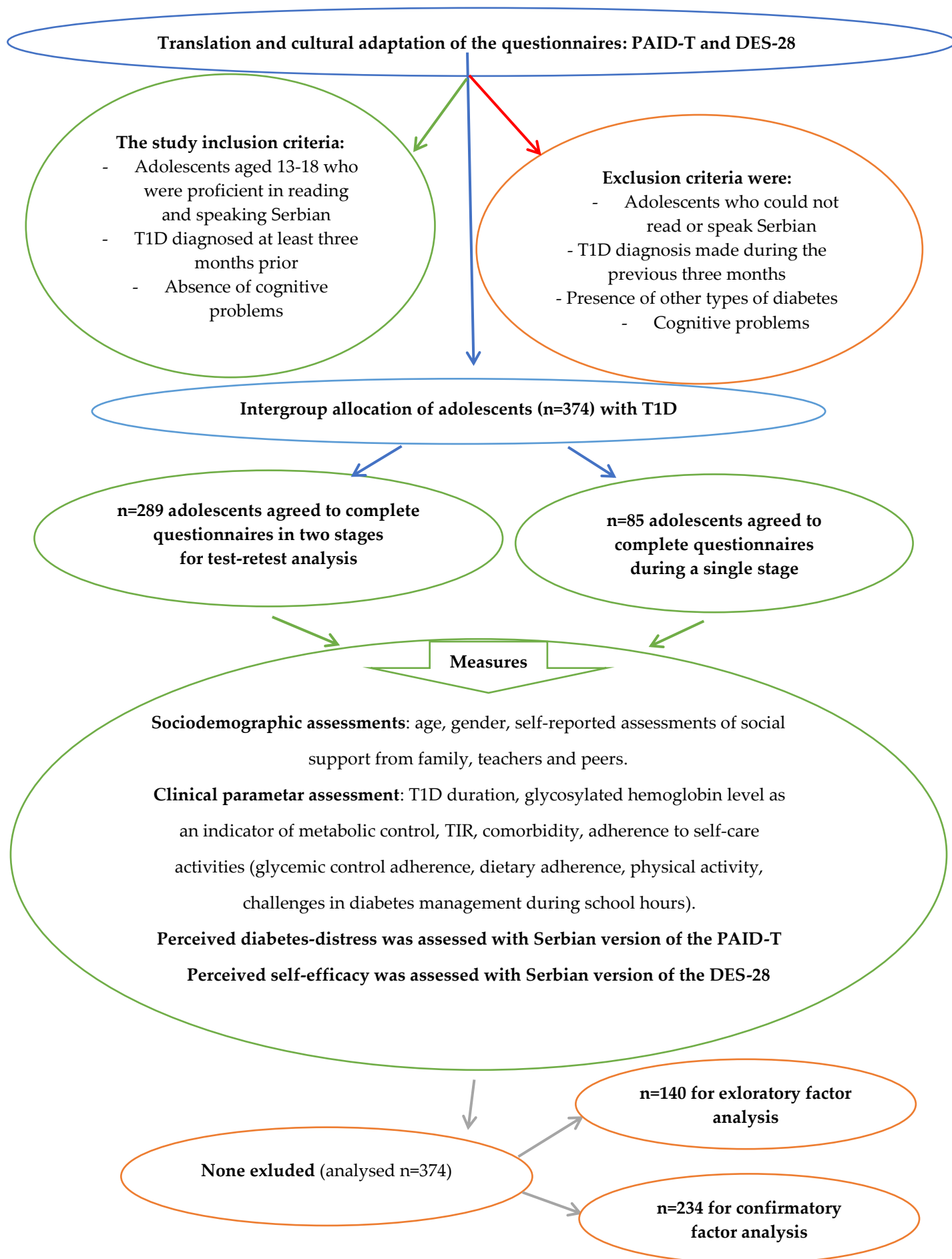

Figure S1. Flow diagram of study subjects

Supplement: Supplementary file 1 [file nursrep-15-00326-s001.zip › Supplementary File S1. Figure S1. Flow diagram of study subjects.pdf]
